# Can a Pressure Standard be Based on Capacitance Measurements?

**DOI:** 10.6028/jres.103.011

**Published:** 1998-04-01

**Authors:** Michael R. Moldover

**Affiliations:** National Institute of Standards and Technology, Gaithersburg, MD 20899-0001

**Keywords:** *ab initio* calculations, cross capacitor, dielectric constant, helium, polarizability, pressure standard, toroidal cross capacitor, molar polarizability

## Abstract

We consider the feasibility of basing a pressure standard on measurements of the dielectric constant *ϵ* and the thermodynamic temperature *T* of helium near 0 °C. The pressure *p* of the helium would be calculated from fundamental constants, quantum mechanics, and statistical mechanics. At present, the relative standard uncertainty of the pressure *u*_r_(*p*) would exceed 20 × 10^−6^, the relative uncertainty of the value of the molar polarizability of helium *A_ϵ_* calculated *ab initio*. If the relativistic corrections to *A_ϵ_* were calculated as accurately as the classical value is now known, a capacitance-based pressure standard might attain *u*_r_(*p*) < 6 × 10^−6^ for pressures near 1 MPa, a result of considerable interest for pressure metrology. One obtains *p* by eliminating the density from the virial expansions for *p* and *ϵ* − 1. If *ϵ* − 1 were measured with a very stable, 0.5 pF toroidal cross capacitor, the small capacitance and the small values of *ϵ* − 1 would require state-of-the-art capacitance measurements to achieve a useful pressure standard.

## 1. Introduction

Recently, there have been remarkable advances in *ab initio* calculations of *ϕ* (*r*), the helium dimer potential [1]. Today, for both ^3^He and ^4^He, the viscosity *η* (*T*), the thermal conductivity *λ* (*T*), and the second virial coefficient *B*(*T*) can be calculated from *ϕ* (*r*) more accurately than they can be measured throughout a wide range of temperatures [2]. Indeed, the *ab initio* results can be used as standards to calibrate instruments designed to measure *η* (*T*), *λ* (*T*), and *B*(*T*). In the present work, we consider both the *ab initio* calculation and the state-of-the-art measurement of the relative electric permittivity *ϵ* (*p*), that is, the “dielectric constant,” as a function of pressure. We conclude that, with plausible advances in the calculations and with state-of-the-art measurements, there is a range of conditions near 1 MPa and 273.16 K such that *ϵ* (*p*) for helium can be calculated more accurately than it can be measured because of the limitations of the piston gage standards used for measuring pressure. If these advances were achieved, the *ab initio* calculation and the measurement of *ϵ* (*p*) could be combined to improve the standards for pressure.

It is not possible to evaluate rigorously all of the uncertainties in the accurate determination of *ϵ* (*p*) for helium until the measurements are attempted. Here we suggest a specific approach to measuring *ϵ* (*p*), identify the primary sources of uncertainty associated with this approach, and estimate their size.

In its essence, the idea is to use the virial equation of state to compute the pressure from the density of the helium and to use capacitance measurements to determine the density. A “working equation” for the pressure can be obtained by eliminating the density *ρ* from the virial expansion for the pressure

$$p=\rho RT\left(1+B\rho +C\rho^{2}+D\rho^{3}+\ldots \right)$$ (1)

and the virial expansion for the dielectric polarizability P

$$P=\frac{\epsilon -1}{\epsilon +2}\frac{1}{\rho }=A_{\epsilon }\left(1+b\rho +c\rho^{2}+\ldots \right).$$ (2)

Here, *B*, *C*, and *D* are the density virial coefficients, *b* and *c* are the dielectric virial coefficients, *A_ϵ_* is the molar polarizability of helium, and *R* is the molar gas constant. One obtains

$$p=\frac{RT}{A_{\epsilon }}E^{*}(1+E^{*}B^{*}+E^{*2}C^{*}+\ldots$$ (3)

with

$$\begin{array}{l} E^{*}=\frac{\epsilon -1}{\epsilon +2};\;B^{*}=\frac{\left(B-b\right)}{A_{\epsilon }};\\ C^{*}=\frac{\left(C-c-2Bb+2b^{2}\right)}{A_{\epsilon }}. \end{array}$$ (4)

In the measurements comtemplated, *ϵ* will be determined from the ratio *C_x_*(*p*)/*C_x_*(0) where *C_x_*(*p*) is the capacitance of a toroidal cross capacitor immersed in helium at a pressure *p* and *C_x_*(0) is the capacitance of the same capacitor when evacuated. The dimensions of the capacitor will decrease under hydrostatic pressure. We assume that this decrease is a linear function of pressure characterized by a coefficient *α_p_*. Upon including this effect, the expression for *ϵ* is:

$$\epsilon \left(p\right)=\frac{C_{x}\left(p\right)}{C_{x}\left(0\right)}\left(1+\alpha_{p}p\right).$$ (5)

Equation (5) implicitly assumes that the mole fractions *x_i_* of any impurities that may be present in the helium are known and accounted for. Equations (3)–(5) comprise a quantitative representation of a hypothetical capacitance-based pressure standard. To discuss systematically the uncertainties of this standard, we consider the pressure as a function of experimentally and theoretically determined quantities:

$$p=p(A_{\epsilon },B,b;R;T,C_{x}(P)/C_{x}(0),\alpha_{p},C,c,x_{i}).$$ (6)

In Eq. (6) and in the discussion below, the quantities are grouped according to how they will be determined. The quantities *A_ϵ_*, *B*, and *b* will be determined from theory; *R* is taken from measurements already made at NIST; *T*, *C_x_*(*p*)/*C_x_*(0), *α_P_*, *C*, *c*, *x_i_* and *p* will be measured to determine the pressure.

## 2. Quantities From Theory and *R*

### 2.1 Molar Polarizability

In 1996, Luther et al. [3] reviewed the calculations of the polarizability *A_ϵ_* of ^4^He in the context of dielectric constant gas thermometry. They noted that the non-relativistic infinite-mass values of *A_ϵ_* obtained in Ref. [4] and in Ref. [5] using different methods agreed to seven significant figures; however, the finite mass and relativistic corrections were not known nearly as well. Luther et al. concluded that

$$A_{\epsilon }=\left(0.517\;253\pm 0.000\;010\right)\;{\text{cm}}^{3}{\text{mol}}^{-1}$$ (7)

and they estimated the relative standard uncertainty *u*_r_ by considering “the order of magnitude of the discrepancies between the published figures for the non-relativistic infinite-mass *A_ϵ_* value and for the corrections to this value, as well as the fact that so far the Lamb shift correction [of order *α*^2^/ln *α*^−1^ (= 11 × 10^−6^)] has not been included.” The value *u*_r_(*A_ϵ_*) = 20 × 10^−6^ is the largest contribution to the uncertainty of the pressure deduced from capacitance measurements. Thus, one purpose of this manuscript is to advocate improvement of these calculations.

### 2.2 Second Density Virial Coefficient

A recent *ab initio* calculation [1] of the helium dimer potential *ϕ* (*r*) claims uncertainties of “0.1 % of the interaction energy, or 0.01 K, whichever was larger” and we take this statement to refer to one standard uncertainty. Hurly and Moldover [6] defined three smooth functions, one that fit the *ab initio* values for *ϕ* (*r*), and two that fit the *ab initio* values incremented and decremented by their uncertainty. These functions were used to compute three sets of values for *B*(*T*) of ^4^He resulting in the value

$$B=\left(11.9059\pm 0.0051\right)\;{\text{cm}}^{3}{\text{mol}}^{-1}$$ (8)

at 273.16 K. The uncertainty indicated in Eq. (8) is the span of the three values which, in turn, reflects the uncertainty of the underlying *ab initio* “data.” The numerical methods used in Ref. [6] to compute *B* from *ϕ* (*r*) have been refined until their contributions to the uncertainties of *B* were negligible. For completeness, we note that Janzen and Aziz [7] used the same *ab initio* “data,” a different functional form for *ϕ* (*r*), and different numerical methods than Hurly and Moldover [6], and obtained a value of *B* that was 0.011 cm^3^ mol^−1^ larger than Eq. (8) and outside the range of combined error estimates. In our opinion, the lower accuracy of the numerical methods used in Ref. [7] account for the different values of *B*.

Using Eq. (8) to evaluate the virial contribution to *p* and its uncertainty, one finds:

$$\begin{array}{c} B\rho \approx Bp/\left(R\times 273.16\;K\right)=\left(524.44\pm 0.22\right)\\ \times 10^{-6}\left(p/\text{bar}\right). \end{array}$$ (9)

At pressures above 20 bar, (1 bar = 0.1 MPa) the uncertainty in *Bρ* will be the most important component of the uncertainty of *p* deduced from capacitance measurements, assuming that *u*_r_(*A_ϵ_*) is reduced by a factor of 5.

### 2.3 Second Dielectric Virial Coefficient

The second and third dielectric virial coefficients of helium, *b* and *c*, are approximately a factor of 100 smaller than the second and third density virial coefficients (*B* and *C*); however, the absolute uncertainties in the two sets of quantities are comparable. The theoretical and experimental results for the second dielectric virial coefficient *b* for helium were reviewed by White and Gugan [8] in 1992. They measured *b* in the range from 3 K to 18 K obtaining the result *b* = (−0.001 ± 0.004) cm^3^ mol^−1^. They reanalyzed the data of Lallemand and Vidal [9] at 298 K and obtained the result *b* = (−0.079 ± 0.012) cm^3^ mol^−1^ when imposing the constraint that *A_ϵ_* should equal its value from *ab initio* calculations. The most recent *ab initio* calculation of *b* cited by White and Gugan [10] was published in 1982, long before the recent advances in calculations. For the present purposes, we simply assume that *b* will be recalculated and that its new uncertainty will be substantially smaller than the 0.005 cm^3^ mol^−1^ uncertainty in *B*. If this is so, the term 0.22 × 10^−6^ (*p*/bar) in Eq. (9) will fully account for the uncertainty in E*B* in Eq. (3).

### 2.4 Molar Gas Constant

The molar gas constant was redetermined in Ref. [11] and has the value

R=(8.314471±0.000014)J/(molK) (10)

with *u*_r_(*R*) = 1.7 × 10^−6^. This relative standard uncertainty contributes the same relative uncertainty to *p*.

## 3. Quantities to be Measured

### 3.1 Temperature

#### 3.1.1 Temperature of the Helium

The capacitance-based pressure standard requires knowledge of the thermodynamic temperature of the gas. If the standard is operated within a few degrees of 273.16 K, the difference between the ITS-90 temperature and the thermodynamic temperature *T* will make a negligible contribution to the uncertainty in the pressure. A temperature uncertainty of 0.3 mK relative to ITS-90 is readily attainable at a specific point within a thermostat and it would contribute only *u*_r_(*T*) = 1 × 10^−6^ to the relative uncertainty in *p*.

A multi-shell, metal thermostat can be designed to house the cross capacitor(s) in an environment where temperature gradients are negligible. To cite one recent example [12], a temperature difference of (5 ± 1) μK was measured between the ends of a 6 cm long, thin-walled, stainless-steel tube maintained slightly below ambient temperature (16 °C).

#### 3.1.2 Temperature of the Capacitor

We assume that the capacitance ratio *C_x_*(*p*)/*C_x_*(0) will be measured with a noise-limited relative standard uncertainty of 2 × 10^−9^ (Sec. 3.2.). This assumption sets the upper bound *α*_T_ × δ*T* ≪ 2 × 10^−9^, where *α*_T_ is the thermal expansion coefficient of the length(s) that determine the capacitance and δ*T* is the uncertainty in measuring the temperature change during the time from the beginning of the measurement of *C_x_*(*p*) to end of the measurement of *C_x_*(0). To allow for thermal equilibration, this interval will be a substantial fraction of an hour or longer. If δ*T* is taken to be 0.3 mK as above, one requires *α*_T_ ≪ 6.7 × 10^−6^ K^−1^. This implies that the capacitances must be defined by materials with lower thermal expansion than the metallic elements or typical alloys. Perhaps invar (*α*_T_ = 1.3 × 10^−6^ K^−1^) or superinvar will be satisfactory if their ferromagnetic properties do not lead to other problems, such as unpredictable frequency or voltage dependencies of the cross capacitance or magnetically-caused microphonic behavior. Alternatively, one could require that δ*T* ≪ 0.1 mK. If this were done, the capacitors could be constructed of conventional materials such as copper or stainless steel. (Evidence for the feasibility of this alternative was published by Furakawa et al. [13]. They made temperature measurements near the triple point of water extending over a period of several days with uncertainties of approximately 0.02 mK.)

### 3.2 Capacitance Measurements

#### 3.2.1 Capacitance Bridge

AC Bridges used for state-of-the-art comparisons of capacitors have remarkable properties [14],[15]. Uncertainties of less than 1 × 10^−9^ are claimed for a wide range of impedances. For the small capacitances (0.5 pF) considered here, noise is a problem. A recent comparison of the NIST 0.5 pF calculable capacitor to a bank of 10 pF capacitors reported that “variability of repeated observations” contributed 2 × 10^−9^ to the relative standard uncertainty of the comparison [16]. We assume that the noise will be no greater when comparing two 0.5 pF capacitors. From Eqs. (1) to (3), one finds (*ϵ*/*p*)(∂*p*/∂*ϵ*) ≈ 1/(*ϵ* − 1). The noise in the capacitance ratio measurements results in a relative standard uncertainty of 2 × 10^−9^/(*ϵ* − 1) ≈ 29 × 10^−6^/(*p*/bar) in the pressure. This uncertainty would be the most important limitation of a capacitance-based pressure standard below 7 bar, assuming that *u*_r_(*A_ϵ_*) is reduced by a factor of 5.

In addition to noise, other effects contribute to uncertainties in capacitance measurements. These include frequency (loading) effects, microphonic coupling, [17] and bridge non-linearities. For the pressure standard considered here, the capacitance ratio varies in the narrow range 1 < *C_x_*(*p*)/*C_x_*(0) < 1.007. Therefore, we assume that the uncertainties resulting from these effects can be made negligible, in contrast with situation encountered when the 0.5 pF NIST calculable capacitor was compared to 10 pF capacitors [16],[18].

#### 3.2.2 Cross Capacitors

**Fig. 1:**
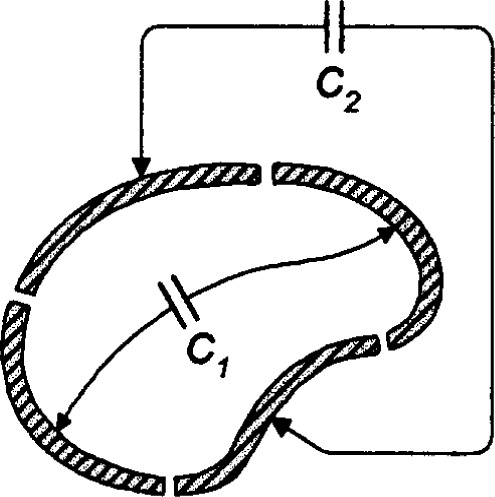
Cross section of four cylindrical conductors comprising a cross capacitor.

In order to make highly accurate measurements of *ϵ* − 1 when *ϵ* − 1 is very small, as it is for dilute helium gas, one requires an extraordinarily stable capacitor. We propose exploiting the remarkable properties of cross capacitors to attain such stability, thereby forgoing the larger capacitance that is available from other geometries. Cross capacitors came to the attention of metrologists when Thomson and Lampard [19] demonstrated that they could be used to make a capacitor whose value could be calculated to high accuracy from a single length measurement. Lampard considered four infinitely-long cylindrical conductors of arbitrary cross section; see Fig. 1. The conductors were separated by thin insulating gaps. Lampard proved [20] that the capacitances per unit length *C*_1_ and *C*_2_ between opposing pairs of conductors obey the relationship:

$$\exp\left(-\pi C_{1}/\epsilon_{0}\right)+\exp\left(-\pi C_{2}/\epsilon_{0}\right)=1$$ (11)

where *ϵ*_0_ = 10^7^/[4π *c*_0_^2^/(m/s)^2^] F/m is the electric permittivity of vacuum and *c*_0_ ≡ 299 792 458 m/s is the defined speed of light. Equation (11) is remarkable because the result applies to all cylindrical conductors, independent of their shapes. Eq. (11) is particularly useful when *C*_1_ ≈ *C*_2_. In this case, it is convenient to define the mean capacitance *C_x_* ≡ (*C*_1_ + *C*_2_)/2 as “the” cross capacitance and to define the difference Δ*C* ≡ (*C*_1_ − *C*_2_). One finds that

$$\begin{array}{c} C_{x}=\frac{\epsilon_{0}\ln\;2}{\pi }\left[1+\frac{\ln\;2}{8}{\left(\frac{\Delta C}{C_{x}}\right)}^{2}+\ldots \right]\\ =1.953\;549\ldots \left[1+0.086\;64\ldots {\left(\frac{\Delta C}{C_{x}}\right)}^{2}+\ldots \right]\frac{\text{pF}}{m} \end{array}$$ (12)

which explicitly shows that *C_x_* depends upon Δ*C* in the second order. This second-order dependence allows one to design cross capacitors that are remarkably insensitive to details of their construction. For many years, this was exploited by national standards laboratories that used evacuated, cylindrical cross capacitors to realize standards of capacitance that were then used to realize the ohm [21]. Despite this history, the author is not aware of any application of cross capacitors either to dielectric constant gas thermometry or, as is advocated here, to accurate measurement of the polarizability of a gas. Most likely, this is a consequence of the very small values of *C_x_* that are readily attainable. The small values of *C_x_* lead to extraordinary demands on the capacitance bridge.

#### 3.2.3 Toroidal Cross Capacitors

**Fig. 2:**
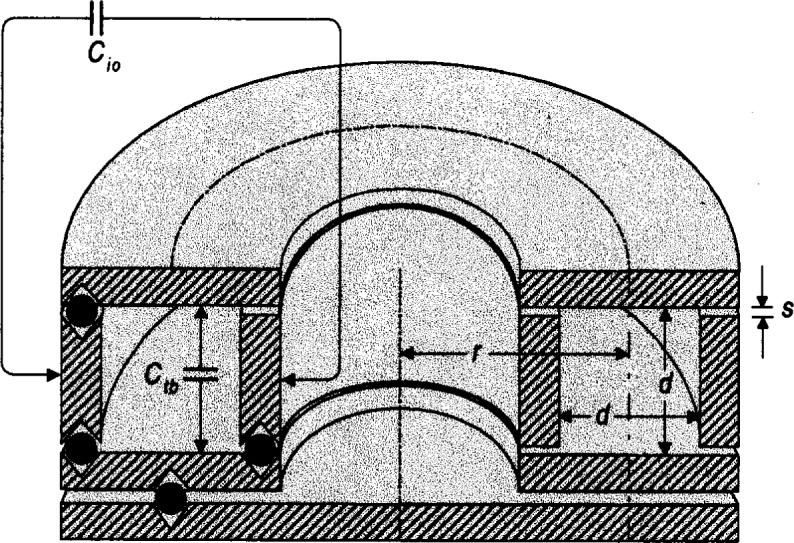
Schematic cross section of a toroidal cross capacitor. The capacitor is rotationally symmetric about the vertical axis shown, except for the insulating balls that support the conductors. For a prototype, the dimensions chosen were: *R* = 50 mm, *d* = 10 mm, and *s* = 0.2 mm. The calculated capacitance between the top and bottom rings is *C*_tb_ = 0.615 pF; the capacitance between the inner and outer cylinders is *C*_io_ = 0.610 pF.

For a hypothetical pressure standard, we consider a toroidal cross capacitor shown in cross section in Fig. 2. This capacitor is assembled from four right circular metal cylinders supported by insulating spheres. Two of the cylinders are disk-like and two could be bored out of bar stock. Thus, this capacitor is comparatively easy to fabricate and it is easy to polish the metal electrodes. Suitable materials for the electrodes and insulators may be invar and glass, respectively, and the properties of these materials will be used for estimates. An evacuated toroidal capacitor, similar to the one shown in Fig. 2, was used for an absolute measurement of loss angle [17]. In Ref. [17], it was shown that the effects of thin dielectric films on the electrodes (such as oxide or oil films) tend to cancel from the cross capacitance. For the toroidal capacitor shown in Fig. 2, the dependence of the cross capacitance on the size of the insulating gaps and on the average radius *r* has been calculated [22]:

$$\begin{array}{c} C_{x}=2\;\ln\;2\;r\epsilon_{0}\epsilon f\left(\frac{d}{r},\frac{s}{d}\right)\\ f\left(\frac{d}{r},\frac{s}{d}\right)=1-0.04042{\left(\frac{d}{r}\right)}^{2}-0.0017{\left(\frac{s}{d}\right)}^{2}+\ldots . \end{array}$$ (13)

As expected, *C_x_* depends, in lowest order, on (*s*/*d*)^2^ where *s* is the width of the gap and *d* is the separation of the electrodes. In the model capacitor shown in Fig. 2, (*s*/*d*) = 0.02 and (∂*C_x_*/∂*s*)/*C_x_* = − 7 × 10^−5^/*d*. Therefore, a small change in *s*, such as that produced by hydrostatic compression of the insulating balls, will have a negligible influence on *C_x_*. In contrast, the corresponding derivative for a conventional, parallel plate capacitor with an insulating gap of thickness *s* is (∂*C_x_*/∂*s*)/*C_x_* = − 1/*s*.

In the work contemplated here, two nearly-identical toroidal cross capacitors would be made and they would be housed in separate, nearly identical pressure vessels. When one of the vessels is evacuated, the capacitor within it would be used as a stable standard of capacitance while the dependence of the second capacitance upon the helium pressure is being measured. Of course the roles of the standard and the test capacitor can be interchanged. Furthermore, as discussed below, the two nearly identical capacitors and enclosures are suitable for using the methods of Burnett [22] and of Buckingham et al. [23] to measure *C*, *c* and the higher terms in Eqs. (1) and (2).

### 3.3 Deformation of Capacitors Under Pressure

The cross capacitances of the toroidal capacitors are proportional to their circumferences (and to the dielectric constant of the surrounding helium); however, they are essentially independent of the properties of the insulating balls, provided that the gaps are sufficiently small and provided that the balls are recessed from the interior of the capacitor. Assuming that the invar conductors are isotropic, hydrostatic pressure decreases their circumferences and *C_x_* by the factor (1 + *α_P_p*), where 3*α_P_* = − (∂*V*/∂*p*)*_T_*/*V* ≡ *k*_T_ is the volumetric isothermal compressibility. For steels and similar alloys, *α_p_* ~ − 0.3 × 10^−6^ bar^−1^.

An accurate value for *k*_T_ (and *α_p_*) of the alloy used to make the electrodes can be determined from the thermodynamic relation

$$k_{T}=-\frac{1}{\rho }\left(\frac{1}{{v_{s}}^{2}}+\frac{{\beta_{p}}^{2}T}{C_{p}}\right)$$ (14)

where *ρ*, *v_s_*, *β_p_*, and *C_p_* are the density, speed of compressional waves, volumetric expansion coefficient, and constant-pressure heat capacity, respectively. Because *β_p_* is so small for invar, the term *β_p_*^2^*T*/*C_p_* in Eq. (14) is only 2 × 10^−4^ of 1/*v*_s_^2^. The quantity *ρ v*_s_^2^ can be determined with a relative standard uncertainty of 10^−3^ by resonant ultrasonic spectroscopy (RUS) [24]. For RUS, one machines a small parallelpiped (typically 3 mm on a side) from the same billet that is used to make the capacitors. The density and the frequencies of the acoustic resonances of the parallelpiped are then measured.

Using Eqs. (3) to (5), one finds in lowest order *p*(∂*α_p_*/∂*p*) = (*ϵ* − 1)/*p* ≈68 × 10^−6^/(*p*/bar). If *α_p_* is measured with a relative standard uncertainty of 10^−3^, the uncertainty of *α_p_* will contribute (10^−3^) × (0.3 × 10^−6^ bar^−1^) ≈4.4 × 10^−6^ to the relative uncertainty in *p*.

This analysis may be considered optimistic because the deformation of conventional capacitors under pressure [8] is more complex than that assumed here. Capacitors are assembled objects, not simply samples of a uniform material. Theoretically, the insensitivity of the cross capacitance to the dimensions of the gaps between the conductors is a strong reason for optimism; however, for standards applications, it will be essential to verify by measurement that cross capacitors of different sizes or designs do deform under pressure as expected.

We note that the other pressure standards (piston gages) also depend upon the dimensions of solids; thus, they too require knowledge of *k*_T_ (or *α_p_*). For example, in the simplest model, the effective area of a piston and cylinder varies as (1 + *α_p_p*) × (geometrical factor) where the geometrical factor mostly depends on the inner and outer diameters of the cylinder. For the capacitance-based pressure standard, the equivalent geometrical factor is amplified by the factor 1/(*ϵ* − 1).

### 3.4 Higher Virial Coefficients

The density and dielectric virial coefficients can be determined by the now-standard experimental methods introduced by Burnett [22] for *B* and *C* and by Buckingham et. al. [23] for *b* and *c*. The former has been used by numerous authors to determine the equation of state of helium and the latter has been recently used for helium in the context of dielectric constant gas thermometry [8].

#### 3.4.1 Dielectric Virial Coefficients

Buckingham et. al [23] recognized that accurate density rather than accurate pressure measurements are required to determine accurately the dielectric virial coefficients. Their method exploits the ability of a symmetric apparatus to halve the density more accurately than the density itself can be determined from Eq. (1). One capacitor is filled with helium at a density *ρ* while the other is evacuated. The two capacitances are measured. The helium is expanded into the second capacitor and the two capacitances are measured again after thermal equilibrium is restored. If both capacitors and their pressure vessels were identical and if the dilations of the pressure vessels and the compressions of the capacitors were linear functions of the pressure, the expansion would very nearly halve the density of the gas. (The density is not exactly halved because of other experimental effects. For example, the valve separating the two pressure vessels will have an asymmetric volume that must be accounted for.) Upon using Eq. (2) twice, once before and once after the expansion, one obtains

$$E^{*}\left(\rho \right)-2E^{*}\left(\rho /2\right)=\frac{\rho^{2}A_{\epsilon }}{2}\left(b+\frac{3c}{2}\rho +O\left(\rho^{2}\right)\right).$$ (15)

If the measurements are repeated using various values of the initial density *ρ*, the separate contributions of *b* and *c* to E*(*ρ*) − 2 E*(*ρ*/2) can be determined. When *b* ≪ *cρ* and *cρ* ≪ *O*(*ρ*^2^) as is the case for *p* < 100 bar, the uncertainty in *cρ* will be comparable to that in *b*. Partial compensation for the asymmetries in the apparatus can be made by repeating the measurements while interchanging the roles of the empty and filled capacitors.

#### 3.4.2 Density Virial Coefficients

The Burnett method of measuring *C* and *D* is similar to Buckingham et al.’s method of measuring *c* and *d* except that pressures rather than capacitances are measured before and after expansions. Conventional measurements determine *B*, *C*, and *D* simultaneously and their uncertainties are correlated. Here, we propose using *ab initio* values for *B* and its uncertainty.

Table 1 displays the relative importance of the terms in Eq. (1) using the values of *B*, *C*, and *D* for helium at 273.15 K from Ref. [25].

**Fig. 3:**
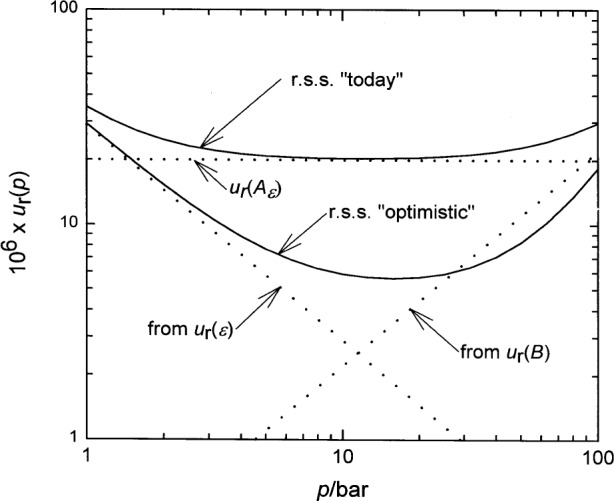
Relative standard uncertainty of the calculated pressure as a function of pressure *u*_r_(*p*). The dotted lines are the contributions to *u*_r_(*p*) from the capacitance ratio measurement [29 × 10^−6^/(*p*/bar)], the *ab initio* second density virial coefficient [0.22 × 10^−6^(*p*/bar)], and *ab initio* calculation of the polarizability *A_ϵ_* (20 × 10^−6^). The upper solid curve (r.s.s. “today”) is the sum in quadrature of these three terms and the other terms in Table 2. The lower solid curve (r.s.s “optimistic”) is the sum in quadrature of all the terms in Table 2, assuming a factor of 5 reduction in *u*_r_(*A_ϵ_*) and a factor of 2 reduction in *u*_r_(*B*) and *u*_r_(*α_p_*).

The data in Table 1 can be compared with a target value of *u*_r_(*p*), for example *u_r_* (*p*)_target_ = 10^−5^. There is a range of pressures such that *u*_r_(*p*)_target_ ≪ *Bρ* ≪ *Cρ*^2^ ≪ *Dρ*^3^. Thus, it appears possible to determine the higher virial coefficients at higher pressures and extrapolate them to the lower pressures where they are needed for the present application and where they cannot be separately determined because of the resolution limits and the dynamic range limits of pressure gages. As a representative example of the degree to which *B*, *C*, and *D*, can be separated, we used the data from Blancett et al. [25]. Their data extend up to 70 MPa and they report *u*_r_(*C*) = 0.022 and *u*_r_(*D*) = 0.084. Ignoring the correlations between Blancett et al.’s values of *B*, *C*, and *D*, the uncertainty in *Cρ*^2^ + *Dρ*^3^ would contribute more than 10^−5^ to *u*_r_(*p*) at pressures above 45 bar, which would limit the usefulness of the capacitance based pressure standard to *p* ≤ 45 bar. We reanalyzed Blancett et al.’s data with the constraint that *B* equal its *ab initio* value. We found that *u*_r_(*C*) = 0.007 and *u*_r_(*D*) = 0.038 and that the virial contribution to *u*_r_(*p*) then reaches 10^−5^ near 80 bar. In Table 2 and Fig. 3, we have used the approximate expressions *u*_r_(*C*)*Cρ*^2^ ≈ 1.4 × 10^−9^ (*p*/bar)^2^ and *u*_r_(*D*)*Dρ*^3^ ≈ 2.5 × 10^−12^ (*p*/bar)^3^ to summarize these results.

We emphasize that the values of *C*, *D*, *u*_r_(*C*) and *u*_r_(*D*) resulting from the present reanalysis are not definitive. They simply indicate that the proposed standard is feasible and that its upper pressure range is sensitive to the quality of the measurements of *C* and *D* and to the assumptions used to analyze the measurements.

### 3.5 Purity of Gas Samples

Because the polarizability of helium is so small compared to other gases, the effects of chemical impurities in helium will be disproportionately large. Gases that have dipole moments, such as water, have much larger temperature-dependent polarizabilities. For water, *A_ϵ_* ≈ [3.71 + 75.49(273 K/*T*)] cm^3^/mol, and near 273 K, one water molecule contributes 153 times as much as one helium atom to *ϵ* − 1. If the mole fraction of water *x*_w_ in helium is to contribute less than 1 × 10^−6^ to the relative standard uncertainty of the capacitance-based pressure standard, *x*_w_ must be known to 6 × 10^−9^. Several manufacturers sell “point of use” helium purifiers that claim to deliver helium with *x*_w_ < 1 × 10^−9^ and similar performance is claimed for other chemically reactive impurities. The requirement for inert impurities is less stringent. For example, the specification is *x*_Ar_ < 1 × 10^−7^ for argon for which *A_ϵ_* = 4.196 cm^3^/mol. It appears that commercially supplied helium treated with a point-of-use purifier will be satisfactory for a capacitance-based pressure standard.

### 3.6 Pressure

Any practical realization of a capacitance-based pressure standard will require a method of separating the carefully purified and thermostated helium in the toroidal cross capacitor from gas in a manifold leading to a conventional apparatus for the measurement of pressure. This separation could be achieved by installing a metal differential pressure indicator (DPI) between the cross capacitor and the external manifold, most likely in the same thermostat as the cross capacitor. A state-of-the-art DPI was developed by Wagner et al. Working near 4 MPa, they measured differential pressures with an “absolute uncertainty in the differential pressure” of less than ± 10 Pa and “scatter” or “consistency” of ± 0.2 Pa. If their design were re-scaled to reduce the working pressure and the “absolute uncertainty” proportionately, it would be barely adequate for the present application. If the proposed capacitance-based pressure standard were operated in such a way that only a small fraction of the full pressure appeared across the DPI, a less elaborate DPI would suffice.

## 4. Conclusions

We return to the question in the title. The terms contributing to the uncertainty in the pressure computed from Eqs. (3) to (5) are listed in Table 2 and the quadrature sum of all the terms is the uppermost solid curve plotted in Fig. 3. The sum is dominated by the uncertainty in the *ab initio* value of *A_ϵ_*. The sum can be compared with the relative standard uncertainty of existing pressure standards which, for NIST, are specific, well-characterized piston gages [27]. The gage PG34, which has an upper limit of 14 bar, has a relative standard uncertainty of 9 × 10^−6^. The gage PG23, which has an upper limit of 173 bar has a relative standard uncertainty of 17 × 10^−6^. If these numbers are compared with Fig. 3, it is clear that the proposed capacitance-based pressure standard is not competitive with piston gages.

There is an important qualification to the comparison of uncertainties for piston gages and the proposed capacitance-based pressure standard. The uncertainties published in Ref. [27] rely on a specific model for the pressure-dependent deformation of the piston-cylinder sets. For the gage PG23, the effective area is assumed to decrease by the fraction 15 × 10^−6^ at 100 bar. Ongoing numerical modeling of the coupled gas flow and elastic distortions in a piston-cylinder set have yielded preliminary evidence that the area change might be 1.5 to 4 times larger than modeled in Ref. [27], depending upon the detailed shape of the gap between the piston and cylinder [28]. This evidence implies that the uncertainties of conventional pressure measurements have been underestimated. A comparison of piston gages with a capacitance-based pressure standard could test the models for the deformation of piston-cylinder sets.

Returning to Fig. 3, the lower solid curve is the sum in quadrature of the terms in Table 2 with the assumptions that the uncertainties in the theoretically derived quantities *A_ϵ_* and *B* are reduced by a factor of 5 and 2, respectively and that the uncertainty in the measured quantity *α*_p_ is reduced by a factor of 2. Under these optimistic assumptions, there is a range of pressures extending from 4 bar to 40 bar in which a capacitance-based pressure standard would have very useful capabilities.

We propose to measure *ϵ* (*p*) as accurately as possible using conventional pressure standards. One output would be a new experimental value of *A_ϵ_* that tests the approximations made in implementing the fundamental theories of quantum mechanics and statistical mechanics. If it happens that the quality of the test of the theory is limited primarily by the quality of the pressure standards, then the measurement program can be inverted so as to define a capacitance-based pressure standard.

## Figures and Tables

**Table 1:** Relative importance of terms in Eq. (1)

*p*/bar	10^4^ *B ρ*	10^4^ *C ρ*^2^	10^4^ *D ρ*^3^
1	5	0.002	
3.2	17	0.02	
10	52	0.2	<0.001
31.6	164	2.1	0.021
100	500	19.6	0.61
316	1433	161	14
1000	3560	992	221

**Table 2:** Contributions to the standard uncertainty of a capacitance-based pressure standard

Quantity	Section	10^6^ × *u*_r_(*p*)
*A_ϵ_*	2.1	20
*B*	2.2	0.22 × (*p*/bar)
*b*	2.3	≪ 0.22 × (*p*/bar)
*R*	2.4	1.7
*T*	3.1	1
*C_x_*(*p*)/*C_x_*(0)	3.2.1	29/(*p*/bar)
*α_p_*	3.3	4.4
*c_ρ_*	3.4.1	≪ 0.22 × (*ρ*/bar)
*C*	3.4.2	1.4 × 10^−3^(*p*/bar)^2^
*D*	3.4.2	2.5 × 10^−6^(*p*/bar)^3^
*x_i_*	3.5	< 1
